# Colorectal liver metastatic disease: efficacy of irreversible electroporation—a single-arm phase II clinical trial (COLDFIRE-2 trial)

**DOI:** 10.1186/s12885-015-1736-5

**Published:** 2015-10-24

**Authors:** Hester J. Scheffer, Laurien G P H Vroomen, Karin Nielsen, Aukje A J M van Tilborg, Emile F I Comans, Cornelis van Kuijk, Bram B. van der Meijs, Janneke van den Bergh, Petrousjka M P van den Tol, Martijn R. Meijerink

**Affiliations:** 1 Department of Radiology and Nuclear Medicine, VU University Medical Center, de Boelelaan 1117, 1081 HV Amsterdam, The Netherlands; 2 Department of Surgery, VU University Medical Center, de Boelelaan 1117, 1081 HV Amsterdam, The Netherlands

**Keywords:** Irreversible electroporation (IRE), Liver neoplasms/secondary, Local, FDG-PET, PET-CT, Tumor ablation, Neoplasm recurrence, Colorectal liver metastases

## Abstract

**Background:**

Irreversible electroporation (IRE) is a novel image-guided tumor ablation technique that has shown promise for the ablation of lesions in proximity to vital structures such as blood vessels and bile ducts. The primary aim of the COLDFIRE-2 trial is to investigate the efficacy of IRE for unresectable, centrally located colorectal liver metastases (CRLM). Secondary outcomes are safety, technical success, and the accuracy of contrast-enhanced (ce)CT and ^18^F-FDG PET-CT in the detection of local tumor progression (LTP).

**Methods/design:**

In this single-arm, multicenter phase II clinical trial, twenty-nine patients with ^18^F-FDG PET-avid CRLM ≤ 3,5 cm will be prospectively included to undergo IRE of the respective lesion. All lesions must be unresectable and unsuitable for thermal ablation due to vicinity of vital structures. Technical success is based on ceMRI one day post-IRE. All complications related to the IRE procedure are registered. Follow-up consists of ^18^F-FDG PET-CT and 4-phase liver CT at 3-monthly intervals during the first year of follow-up. Treatment efficacy is defined as the percentage of tumors successfully eradicated 12 months after the initial IRE procedure based on clinical follow-up using both imaging modalities, tumor marker and (if available) histopathology. To determine the accuracy of ^18^F-FDG PET-CT and ceCT, both imaging modalities will be individually scored by two reviewers that are blinded for the final oncologic outcome.

**Discussion:**

To date, patients with a central CRLM unsuitable for resection or thermal ablation have no curative treatment option and are given palliative chemotherapy. For these patients, IRE may prove a life-saving treatment option. The results of the proposed trial may represent an important step towards the implementation of IRE for central liver tumors in the clinical setting.

**Trial registration:**

Trial registration number: NCT02082782.

## Background

Colorectal cancer causes each 10th death due to cancer in Western countries. About 33 % of all patients with colon cancer have liver-only metastatic disease [1]. For these patients, surgical resection is the gold standard, with 5-year survival rates up to 60 % [2, 3]. However, despite improvements of surgical techniques and current neoadjuvant chemotherapies, only 5–20 % of patients with colorectal liver metastases (CRLM) can benefit from surgery due to number, localization, or distribution of tumors [4].

In light of the limitations of surgical resection for many hepatic tumors, a number of ablative technologies for liver-directed therapy have developed during the last 20 years [5]. Of these techniques, thermal ablation using radiofrequency ablation (RFA) and microwave ablation (MWA) are most frequently used. A recent review showed a local recurrence rate of 2.8–29.7 % of RF-ablated liver lesions at 12–49 months follow-up, and 2.7–12.5 % of MW-ablated lesions at 5–19 months follow-up, with a 5-year survival rate around 30 % for both techniques [6]. Although thermal ablation has dramatically improved survival rates for patients with CRLM, factors like size and location limit its use and effectiveness. Efficacy of RFA rapidly decreases for lesions > 3 cm [7]. Also, the rate of complete tumor necrosis falls below 50 % when vessels larger than 3 mm abut the tumor as a consequence of the heat-sink effect [8]. Ablation of lesions close to vital structures like major bile ducts and vessels is associated with a substantial risk of complications due to thermal damage.

Irreversible electroporation (IRE) is a novel ablation modality that may overcome some of the limitations of current thermal ablation therapies. It is based on a pulsating current created between multiple needle electrodes placed around the target lesion which alters the existing cellular transmembrane potential. If the duration of the applied electrical pulses is below the charging time of the outer cell membrane, an interaction of the electric field with subcellular structures occurs [9]. This interaction results in permanent permeabilization of the cell membrane, which disrupts cellular homeostasis and ultimately leads to cell death. The irreversibly damaged cells are removed by the immune system [10]. Two main factors stimulate research into IRE as an ablation modality. Since the mechanism of cell death is predominantly nonthermal, connective tissue structure is preserved, so there is no damage to associated blood vessels and bile ducts [11–13]. For the same reason, treatment efficacy is not impeded by heat-sink [14]. This allows for treatment of liver tumors deemed unresectable or ineligible for other focal ablation techniques due to localization near these structures [15].

The capability of IRE to destroy CRLM in humans was recently demonstrated in the COLDFIRE-1 ablate-and-resect trial [16]. In this trial resectable CRLM were treated with IRE, followed by resection one hour later. The investigators demonstrated cell death of the ablated tumors within one hour after IRE, with intact larger vascular and ductal structures within the ablation zone. The first studies investigating hepatic IRE on clinical indication also yield promising results. A systematic review found an overall complication rate of 16 % for hepatic IRE, similar to RFA [17]. However, the tumors treated with IRE were all located near thermally sensitive structures, as opposed to the thermally ablated lesions. Complete tumor eradication was achieved in 67–100 %, and this percentage was even higher for tumors < 3 cm [17]. However, since most studies are retrospective with short-term follow-up using heterogeneous study populations and design, the value of the current evidence remains limited. Also, the optimal imaging modality for follow-up after IRE needs to be analyzed.

The primary aim of the COLDFIRE-2 trial is to investigate the efficacy of IRE for CRLM that are unsuitable for resection and thermal ablation due to the vicinity of vulnerable structures such as bile ducts, vessels and bowel. Other outcomes are safety of IRE, and the accuracy of contrast-enhanced computed tomography (ceCT) and ^18^F-fluorodeoxyglucose (FDG) positron emission tomography (PET) CT in the detection of local tumor progression (LTP).

## Methods/design

### Design

The COLDFIRE-2 trial is a phase II, multicenter, prospective single-arm trial that is organized by the VU University Medical Center in Amsterdam, the Netherlands. Patients will be recruited at three academic hospitals in the Netherlands (VU University Medical Center, Amsterdam; Academic Medical Center, Amsterdam; Leiden University Medical Center, Leiden). The study protocol has been approved by the Medical Ethical Review Board of the VU University Medical Center. The trial is investigator-sponsored, independent of industry and is registered at clinicaltrials.gov under number NCT02082782. The trial will be conducted in accordance with the Declaration of Helsinki. The inclusion and exclusion criteria for the COLDFIRE-2 trial are summarized in Table 1.

**Table 1:** In- and exclusion criteria

Inclusion criteria	Exclusion criteria
Histologic proof of primary colorectal tumor	Ventricular cardiac arrhythmias
Radiologic proof of colorectal liver metastasis, unsuitable for resection and thermal ablation due to vicinity to vascular or ductal structures	Congestive heart failure, NYHA Class ≥ 3
^18^F FDG-PET avidity of target lesion and visible on ceCT	Active coronary artery disease
Lesion size ≤ 3.5 cm	History of epilepsy
Adequate bone marrow, liver and renal function	Any implanted stimulation device
ASA-classification 0–3	Chemotherapy <6 weeks prior to treatment

### Eligibility criteria

All patients are treated with curative intent and must have received previous chemotherapy for CRLM at some stage in their disease. Prior to inclusion, all patients will be discussed in a multidisciplinary liver tumor board consisting of a hepatogastroenterologist, hepatobiliary surgeon, medical oncologist, radiation oncologist, abdominal and interventional radiologist. Decision on treatment will be at their discretion. Patients who present with more than one metastasis can only be included if treatment with curative intent is still realistic and if all lesions can be treated during the same session or within six weeks after the IRE procedure. Thermal ablation or resection of concomitant lesions during the same session is therefore allowed and limited extrahepatic disease, defined as ≤5 nodules in the lung and/or one other metastatic site which is amenable to future definitive treatment, is not contra-indicated [18]. All participants from all participating centers will provide written informed consent.

### IRE procedure

**Fig. 1:**
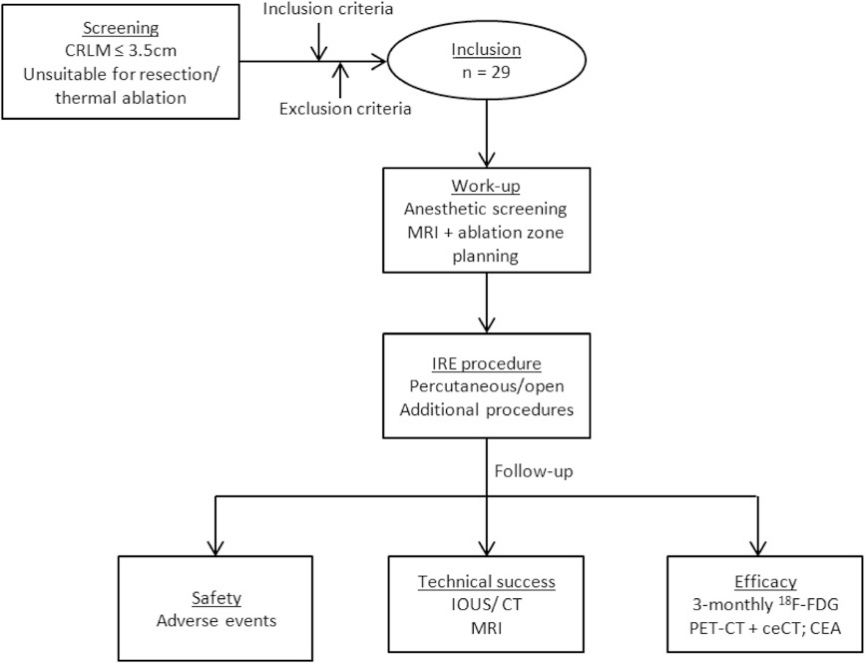
Flow diagram of study procedure

Depending on concurrent treatment of other lesions (surgical resection or thermal ablation), patients will either be treated during an open procedure using intra-operative ultrasound (IOUS), or percutaneously using CT. Patients undergoing laparotomy will receive a thoracic epidural before surgery. For percutaneous procedures, to allow repeated and real-time visualization of both the tumor and the adjacent vessels, a catheter is placed within the common hepatic artery approaching from the right femoral artery. Technical details of this procedure have been previously described by Van Tilborg et al. [19]. All procedures will be performed under general anesthesia, induced with propofol, sufentanil, and rocuronium and maintained with propofol and remifentanil. First, the exact three-dimensional measurements of the target lesion are defined using IOUS or aortic catheter based CT. The planned electrode configuration must result in an expected geometry of the ablation zone that fully covers the tumor and a tumor-free margin of at least 5 mm in all directions [20]. Depending on the size, 2–6 needle electrodes (NanoKnife, AngioDynamics, Latham, NY) with an active working length of 20 mm are positioned in the outer border or just adjacent to the tumor under IOUS or CT guidance, aiming at an inter-electrode distance of 20 mm (±2 mm). All needles are placed parallel to each other to promote homogeneous energy delivery. After confirmation of correct distances with IOUS or with unenhanced CT using multiplanar reformatting, ten test-pulses of 1500 V/cm and 90 μs are delivered via each electrode pair, after which the delivered current is verified. The target current lies between 20–50A and voltage settings are manually adjusted in case of over- or undercurrent. Subsequently, three cycles of 30 pulses are administered to reach a total of 100 pulses per electrode pair. If more than 6 electrodes are needed for larger tumors, electrodes are repositioned to perform overlapping ablations. Similarly, for tumors with a depth larger than 20 mm, after ablation of the deepest part of the tumor a 1.5 cm pullback of the electrodes is performed to treat the superficial part. Immediately after IRE, a control aortic catheter CT or IOUS is made to evaluate technical success and to exclude early complications. The next day, ceMRI is performed to verify patency of vascular and ductal structures, as well as technical success. In case of incomplete ablation on ceMRI, the suspected residual tumor will be retreated in the following weeks. A flow diagram of the trial is shown in Fig. 1.

### Imaging

Four-phase liver CT and ^18^F-FDG PET-CT will be performed before IRE, and 3, 6, 9 and 12 months after IRE. Imaging is conducted according to EANM guidelines with the same ^18^F-FDG PET-CT in each participating center (Philips Gemini TF PET-CT system, Philips Medical Systems, Cleveland, Ohio, USA) [21]. Whole body ^18^F-FDG PET (skull base to mid-thigh) starts 60 min after FDG injection, followed by a low-dose CT for attenuation correction and anatomical co-localization of ^18^F-FDG PET-findings. Next, a diagnostic 4-phase CT of the liver is performed. Scanning parameters are shown in Table 2.

**Table 2:** Scanning parameters of 18 F-FDG PET-CT and 4-phase liver CT

	Delay (sec)	Contrast	Matrix	Pixel size (mm^2^)	Slice thickness (mm)	Current (mAs/slice)	Energy (keV)
Whole body PET		^18^F FDG (ref)	144 × 144	4 × 4	5		
Low-dose CT		-	512 × 512	1.17 × 1.17	5	30–50	100
4-phase liver CT		100 ml	512 × 512	0.68 × 0.68	4–5		
- Precontrast		Xeneti × 300				220 AEC	120
- Arterial	12					450 AEC	80
- Venous	50					220 AEC	120
- Hepatic	220					175 AEC	120

*AEC*; Automated exposure control

Treatment response is primarily based on a per-lesion analysis. The standard of reference is defined by a combination of 4-phase liver CT and ^18^F-FDG PET-CT, and, if available, histologically proven malignancy. Pathologically elevated carcinoembryonic antigen (CEA) is used when appropriate (e.g. if no other metastases are present).

Treatment response on 4-phase liver CT is assessed using the Response Evaluation Criteria In Solid Tumors (RECIST) [22]. The Positron Emission tomography Response Criteria In Solid Tumors (PERCIST) criteria are used for ^18^F-FDG PET-CT [23]. On 4-phase liver CT, LTP is defined as a growing (>20 %; longest diameter; axial plane) hypodense lesion within 1 cm of the ablation zone. On ^18^F-FDG PET-CT, focally increased FDG uptake within 1 cm of the ablation region is considered an LTP [24]. If LTP is suspected on both modalities during follow-up, the patient will be evaluated for re-treatment in our multidisciplinary liver tumor board. To establish the value of PET-CT and ceCT for the prediction of LTP the suspected area will be biopsied in the same session prior to re-treatment, although the suspicious lesion will be re-ablated regardless. In case of discrepancy between the 4-phase liver CT and ^18^F-FDG PET-CT findings, a liver MRI will be performed as problem solver. If MRI is also inconclusive, multidisciplinary deliberation will decide upon either repeat imaging 3 months later, or biopsy of the suspected recurrence. If chemotherapy is indicated during follow-up (e.g. due to new metastatic disease), the patient will remain in follow-up. The trial will end twelve months after the last IRE procedure. If no LTP or new metastatic disease has been diagnosed at this moment, regular follow-up will be resumed consisting of 6-monthly ^18^F-FDG PET-CT and 4-phase liver CT.

### Data collection and handling

The study coordinators (HS and LV) will collect the data. All data will be handled confidentially and anonymously. A subject identification code is used to link the data to the subject. The study coordinators safeguard the key to the identification code. The handling of personal data will comply with the Dutch Personal Data Protection Act.

To investigate the accuracy of ^18^F-FDG PET-CT and 4-phase liver CT in the diagnosis of LTP after IRE, twelve months after the last IRE treatment blinded data sets from all centers will be reviewed separately and independently by two designated radiologists (BvdM and JvdB) and two nuclear medicine physicians (OH and EC) in a consensus reading. Each lesion will be scored on a separate form on a one to five scale (Table 3) [25]. The reviewers will be blinded to the results of the other imaging modality and to the final oncologic outcome as determined by the standard of reference.

**Table 3:** Study form for reviewers’ results (per lesion)

Study number		Reviewer number
Score	Definition	Explanation
1	Normal	Confident that no tumor recurrence is present in the ablation zone
2	Probably benign	The appearance of the ablated lesion is compatible with post-ablational inflammation or rim-like characteristics
3	Equivocal	There is doubt whether the imaging features indicate local tumor progression or inflammation
4	Probably malignant	Confident about local progression in the ablation zone
5	Impaired quality	Quality of the images precludes adequate diagnosis
Comments:		

### Primary and secondary objectives

The primary endpoint of the COLDFIRE-2 trial is efficacy of IRE for CRLM. Secondary endpoints are progression-free survival (PFS) and overall survival (OS). Other secondary endpoints are safety, technical success, and the accuracy of ^18^F-FDG PET-CT and 4-phase liver CT in the detection of LTP after IRE.

Primary efficacy rate is defined as the percentage of target tumors successfully eradicated 12 months after the initial IRE procedure, according to the RECIST and PERCIST criteria. Secondary efficacy rate is defined as the percentage of tumors successfully eradicated 12 months after the initial IRE procedure, including tumors that have undergone successful repeat ablation following identification of LTP [26].

Progression-free survival is defined as the time from the first IRE procedure to the time of radiologic disease progression or death of disease. Overall survival is defined as the time from the IRE procedure until death of disease [27]. Both PFS and OS will be determined using the Metabolic Imaging And Marker Integration (MIAMI) criteria as proposed by Hosein and colleagues, which integrates anatomic response parameters (4-phase liver CT) with two functional parameters: PET activity and CEA levels [28]. For safety analysis, all major adverse events and all adverse events occurring during or within 12 months after IRE treatment will be recorded according to the Common Terminology Criteria of Adverse Events (CTCAE) v4.0 [29]. Pain assessment using the visual analogue scale (VAS) and patient analgesic consumption will be recorded. Patency of vessels and bile ducts on follow-up cross-sectional imaging will be analyzed to identify late complications. Technical success is defined as (1) the ability to successfully deliver all planned pulses according to protocol and (2) complete lesion coverage on post-procedure ceCT or IOUS, and 24–48 h post-procedure MRI [26].

### Sample size calculation and statistical considerations

Based on the current literature our hypothesis is that 10 % of the treated tumors will recur after the initial treatment (primary technique efficacy 90 %), which implies a local recurrence rate of 0.1 [5, 30]. Choosing a target width of 0.25 with p ≤ 0.05, we used the confidence interval formula “Exact” for two-sided confidence intervals for one proportion (Clopper-Pearson) [31, 32]. With this formula, actual width is 0.249, with a 95 % confidence interval of 0.020–0.269. This calculation results in a sample size of 29 patients. Considering a 5 % loss to follow-up, the total number of patients needed is 31.

All clinicopathological and procedural variables will be described and analyzed. Continuous variables will be summarized with standard descriptive statistics including means, standard deviations, medians and ranges. Categorical variables will be summarized with frequencies. *P*-values ≤ 0.05 will be considered statistically significant. Univariate survival analysis will be performed using the Kaplan-Meier method. Differences in local recurrence rate between subgroups like tumor size and adjuvant chemotherapy will be analyzed descriptively since the expected number of recurrences is too small for statistical analysis.

The accuracy of ^18^F-FDG PET-CT and 4-phase liver CT in diagnosing local recurrences will be investigated by comparison to the reference standard. Interobserver-variability will be determined using Cohen’s Kappa. Sensitivity and specificity of both techniques will be calculated with their respective 95 % confidence intervals. McNemar’s test is used to assess a statistically significant difference between both imaging modalities.

## Discussion

### IRE for colorectal liver metastases

New cancer treatments are typically best defined from phase III randomized trials comparing the investigated therapy with the current standard. However, in the field of local tumor ablation, this has proven a difficult challenge. Since its introduction decades ago, the number of randomized trials remains limited. An attempt to organize a trial comparing RFA to surgical resection has failed (French FFCD 2002–02) and it is unlikely that another trial can be organized in the near future [33]. The current literature on the clinical application of IRE is scarce with no randomized controlled trials. However, because the indication for IRE lies with tumors in which no other suitable local therapy is available, a randomized trial comparing IRE to e.g. surgical resection or thermal ablation is not unrealistic at this point in time.

Results of single-arm studies on hepatic IRE report a high variation in efficacy, with local recurrence rates between 67–100 % [17]. Similar to RFA, efficacy is higher for tumors ≤ 3 cm. However, since IRE is only used as a ‘last resort’ curative treatment option in patients that would otherwise receive palliative chemotherapy, these early efficacy rates are promising and encourage larger prospective studies.

### Feasibility

Intraprocedural monitoring and control of ablation play a critical role in the success of tumor ablation [34]. The feasibility of real-time monitoring with US during hepatic IRE has been demonstrated in both animal and human studies. The ablation zone immediately appears as a hypoechoic area with well-defined margins [16, 35, 36]. Immediate postprocedure ceCT also shows a well-defined hypodense area on the portal venous phase with variable periablational hyperenhancement [37, 38]. The size and shape of the IRE ablation zone on both US and CT has proven to correlate reliably with the pathologically defined zone of cell death [36, 37]. These imaging modalities could therefore be used to ensure that the realm of ablation encompasses the originally targeted volume with a sufficient margin [15], which is a focus of the presented trial.

### Imaging follow-up

The main concern following tumor ablation is the risk of developing LTP. Early diagnosis of LTP is imperative because repeated treatment can still effectuate complete tumor clearance, especially for smaller recurrences. CeCT is the current mainstay of staging and follow-up [39]. One shortcoming of ceCT in the monitoring of post-ablative lesions for recurrent disease is the presence of post-ablation effects. Because reactive tissue and viable tumor can both present as hypodensity around the ablated lesion, their distinction can be difficult [24]. Due to the visualization of increased glucose metabolism in tumor cells, ^18^F-FDG PET has proven a very sensitive and accurate tool for the diagnosis of tumor manifestations in patients with colorectal carcinoma [39]. Using ^18^F-FDG PET-CT, ^18^F-FDG PET images are combined with CT in a single session. Anatomical and morphological information from CT is used to increase the precision of localization, extent, and characterization of lesions detected by ^18^F-FDG PET [21]. Several studies have shown the superiority of ^18^F-FDG PET-CT over morphologic imaging alone in the follow-up after ablation of CRLM: sensitivity and specificity for the detection of LTP are 92 % 100 % for ^18^F-FDG PET-CT compared to 83 % and 100 % for ceCT [24]. However, the diagnostic accuracy of ^18^F-FDG PET and ^18^F-FDG PET-CT is strongly affected by chemotherapy, so for patients receiving chemotherapy during follow-up after IRE ceCT may prove the most reliable imaging modality.

After IRE, immediate ceCT shows a hypodense ablation zone that does not enhance post-contrast. A transient peripheral rim of contrast enhancement can be present, representing hyperemia [15]. In the months after ablation, the ablation zone slowly decreases in size and should not show uptake of contrast. PET scans show a dynamic response to the IRE ablation. Three days following IRE, an ^18^F-FDG-avid peripheral zone surrounding the ablated region appears. This initial increase in uptake at the periphery of the IRE region may be explained by an inflammatory response, increasing metabolic activity at the targeted region as the cellular debris is removed from the ablation site [15]. In our experience the inflammatory response can persist for several months, which renders evaluation of the ablation zone with ^18^F-FDG PET difficult. On the contrary, ablated lesions that show focal rather than rim-like uptake in the periphery are considered suspect for local recurrence. Because much is still unknown about the imaging characteristics of liver lesions treated with IRE, standardized follow-up regimens are lacking. The COLDFIRE-2 trial focuses on the typical imaging characteristics of electroporated CRLM over time. The gold standard for LTP is histologic confirmation. With biopsies taken from all suspicious lesions prior to re-treatment, the trial also assesses the accuracy of the pre-defined definition of LTP on PET-CT and ceCT.

A potentially curative treatment option for a group of patients that is currently offered chemotherapy with palliative intent is of major importance. IRE may prove a safe and valuable fortification in the armory of interventional oncologists treating patients with liver tumors. The aim of the COLDFIRE-2 trial is to contribute to the available evidence on IRE with respect to safety, efficacy, imaging characteristics and follow-up guidelines. The results of the proposed trial may represent an important step towards the implementation of IRE for central liver tumors in the clinical setting.

## Abbreviations

ASA: American Society of Anesthesiologists
CEA: Carcinoembryonic antigen
ceCT: Contrast-enhanced computed tomography
CRLM: Colorectal liver metastases
CTCAE: Common terminology criteria of adverse events
^18^F-FDG: ^18^F-fluorodeoxyglucose
IOUS: Intra-operative ultrasound
IRE: Irreversible electroporation
LTP: Local tumor progression
ceMRI: Contrast enhanced MRI
MWA: Microwave ablation
NYHA: New York Heart Association
PET: Positron emission tomography
RFA: Radiofrequency ablation
VAS: Visual analogue scale
